# Amphetamine Administration into the Ventral Striatum Facilitates Behavioral Interaction with Unconditioned Visual Signals in Rats

**DOI:** 10.1371/journal.pone.0008741

**Published:** 2010-01-15

**Authors:** Rick Shin, Junran Cao, Sierra M. Webb, Satoshi Ikemoto

**Affiliations:** Behavioral Neuroscience Research Branch, National Institute on Drug Abuse, National Institutes of Health, United States Department of Health and Human Services, Baltimore, Maryland, United States of America; The Mental Health Research Institute of Victoria, Australia

## Abstract

**Background:**

Administration of psychomotor stimulants like amphetamine facilitates behavior in the presence of incentive distal stimuli, which have acquired the motivational properties of primary rewards through associative learning. This facilitation appears to be mediated by the mesolimbic dopamine system, which may also be involved in facilitating behavior in the presence of distal stimuli that have not been previously paired with primary rewards. However, it is unclear whether psychomotor stimulants facilitate behavioral interaction with unconditioned distal stimuli.

**Principal Findings:**

We found that noncontingent administration of amphetamine into subregions of the rat ventral striatum, particularly in the vicinity of the medial olfactory tubercle, facilitates lever pressing followed by visual signals that had not been paired with primary rewards. Noncontingent administration of amphetamine failed to facilitate lever pressing when it was followed by either tones or delayed presentation or absence of visual signals, suggesting that visual signals are key for enhanced behavioral interaction. Systemic administration of amphetamine markedly increased locomotor activity, but did not necessarily increase lever pressing rewarded by visual signals, suggesting that lever pressing is not a byproduct of heightened locomotor activity. Lever pressing facilitated by amphetamine was reduced by co-administration of the dopamine receptor antagonists SCH 23390 (D1 selective) or sulpiride (D2 selective).

**Conclusions:**

Our results suggest that amphetamine administration into the ventral striatum, particularly in the vicinity of the medial olfactory tubercle, activates dopaminergic mechanisms that strongly enhance behavioral interaction with unconditioned visual stimuli.

## Introduction

Administration of psychomotor stimulants like amphetamine and cocaine facilitates action in the presence of incentive stimuli (called incentive motivation) or action rewarded by conditioned reinforcers (known as conditioned reinforcement). These stimuli are usually understood as conditioned distal cues that have acquired the motivational properties of primary rewards (e.g., nutrients contained in food, brain stimulation reward and drug rewards) through associative learning [1]–[3]. Psychomotor stimulants' ability to facilitate action in conjunction with conditioned incentive stimuli appears to be mediated by the mesolimbic dopamine projections from the ventral tegmental area to the ventral striatum [2], [4]–[9]. This notion is supported by the finding that injections of amphetamine into the nucleus accumbens facilitate action in the presence of or rewarded by conditioned incentive stimuli [10]–[12], whereas 6-hydroxydopamine lesions of the accumbens reduce these actions [13].

It was discovered several decades ago that some distal stimuli - like visual signals - are naturally salient, reinforcing actions without being conditioned with primary rewards. Laboratory animals including rats, mice and monkeys learn to instrumentally respond to obtain presentation of unconditioned light illumination [14]–[17]. Both lever presses and exploration (in novel chambers) maintained by unconditioned stimuli are readily disrupted by systemic treatments of low doses of dopamine receptor antagonists [18]–[20] and by more selective manipulations of 6-hydroxydopamine lesions of the ventral striatum [21], [22] or dopamine receptor antagonist injections into this area [23]. Thus, behavioral interaction with unconditioned stimuli appears to depend on an intact mesolimbic dopamine system [24]. Consistent with this hypothesis, midbrain dopamine neuron activity is increased by both unexpected presentation of unconditioned distal stimuli and conditioned stimuli [25]–[27]. Similarly, extracellular dopamine concentrations in the ventral striatum increase in response to novel or conditioned stimuli [28]–[31].

These findings led us to hypothesize that psychomotor stimulant administration into the ventral striatum, which would increase extracellular dopamine concentrations, would enhance behavioral interaction maintained by unconditioned salient stimuli. However, prior evidence offers mixed support for such a role of psychomotor stimulants. Supporting this hypothesis, rats in “novel” environments increase locomotor activity much more vigorously than those in “home” environments following systemic administration of amphetamine, even when the novel and home environments are physically identical [32], [33]. Conversely, upon closely examining rats' behavioral activity, researchers found that systemic administration of amphetamine or other psychomotor stimulants markedly increased locomotion without facilitating investigation of novel stimuli [18], [34]–[36].

We sought to shed light on this issue by examining amphetamine injections into subregions of the ventral striatum in an operant procedure where responses are rewarded by unconditioned visual signals or tones. This method is modified from previous intracranial self-administration procedures [37], [38] and a similar light seeking procedure described for nicotine administration [39], [40]. We found that when psychomotor stimulants were selectively administered into the ventral striatum, particularly in the vicinity of the medial olfactory tubercle, they facilitate behavioral interaction with unconditioned visual signals, but not tones. In light of previous findings that the medial olfactory tubercle mediates psychomotor stimulants' rewarding effects [37], [38], [41], we then examined the contribution of visual signals on amphetamine self-administration into this subregion.

## Results and Discussion

### Experiment 1: Effects of Injection Sites on Lever Pressing

**Figure 1:**
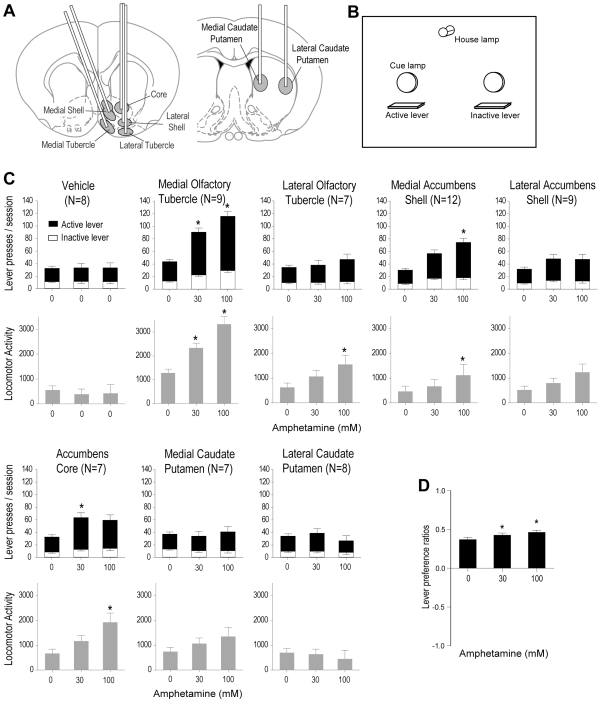
Effects of noncontingent injections of amphetamine into subregions on lever pressing and locomotor activity. A. Schematic drawings showing striatal subregions into which amphetamine was unilaterally injected. B. The arrangement of levers and lamps on a chamber wall is shown schematically. Upon active lever pressing, rats received an illumination of the cue lamp just above the lever for 1 sec, and an extinction of the house lamp for 7 sec, during which lever pressing was counted, but produced no additional visual signal. Responding on the inactive lever had no programmed consequence. C. Rats received vehicle infusions (100 nl per infusion) in sessions 1 and 6, 30 and 100 mM amphetamine in sessions 2–3 and 4–5, respectively, except one group that received vehicle injections into the medial tubercle in all sessions. The data are mean responses on the active (visual signal) lever stacked on inactive (no visual signal) lever presses with SEM over two sessions. Locomotor activities were detected via the movements of the electronic swivel for each rat's infusion pump. * *P*<0.05, significantly greater than vehicle values. D. Preference for the active lever over the inactive lever slightly increased as a function of amphetamine concentration, with all groups collapsed together. The data are mean lever preference ratios with SEM. * *P*<0.05, significantly greater than vehicle values. Formula: lever preference ratio = (active lever presses – inactive lever presses)/(active lever presses+inactive lever presses). This formula produces values ranging between 1 and -1; 0 indicates no preference (i.e., equal numbers of responding between the active and inactive levers), while 1 indicates a complete preference for the active lever (i.e., all responses are made on the active lever).

We compared the effects of noncontingent administration of amphetamine into seven subregions within the striatal complex (Fig. 1A) on lever pressing contingently followed by visual signals (Fig. 1B). Noncontingent amphetamine increased lever pressing when injected into the medial olfactory tubercle, medial accumbens shell and accumbens core, but not the other subregions studied (Fig. 1C; significant interaction between effects of subregion and concentration, *F*_14,116_ = 4.81, *P*<0.0001; the data were analyzed with a mixed ANOVA/MANOVA with the subregion (seven subregions plus vehicle) as between-subjects factor and concentration (3), session (2) and lever (2) as within-subjects factors; the results of the dorsomedial shell and ventromedial shell were combined because they did not differ significantly). The medial olfactory tubercle was most responsive to amphetamine, mediating lever presses more strongly than any other subregion (main effect of subregion, *F*_7,59_ = 7.93, *P*<0.0001 followed by Newman-Keuls test, *Ps*<0.002). The number of lever presses incited by administration of the highest concentration of amphetamine into the medial tubercle was significantly greater than the number of presses occurring when the same amphetamine concentration was injected into any other subregion (*Ps*<0.05), whereas lever presses of vehicle injections into the medial tubercle did not differ from those of vehicle into the other subregions. Additional information including photomicrographs and effectiveness of individual injection sites is presented in Figure S1.

We also examined whether preference ratios of the active lever over the inactive lever changed as a function of concentration (see the formula described in the legend of Fig. 1D). This measure potentially indicates the rewarding effect of stimuli associated with the active lever, distinguished from exploration or general arousal that may have been elicited by the manipulations; in this case, injections of different amphetamine concentrations. Because amphetamine injections into the ventral striatum are known to elicit arousal effects [42], it is important to have a ratio measure that distinguishes lever preference from arousal effects due to the manipulation. Although we found a significant interaction between lever and concentration on lever presses, *F*_2,58_ = 22.14, *P*<0.0001 with the ANOVA/MANOVA described above, it is difficult to interpret this interaction. The significant interaction could arise from amphetamine's ability to increase response on both levers without increasing preference for the active lever over the inactive lever. In other words, amphetamine could increase the difference in response rates between the two levers by the same proportion, leading to a significant lever x manipulation interaction. Our lever preference analysis revealed that amphetamine administration increased preference for the active lever as its concentration increased (main effect of concentration, *F*_2,104_ = 6.69, *P* = 0.002 with an 8×3×2 mixed ANOVA/MANOVA with subregion, concentration and session on lever-preference ratios). However, when lever preference ratios were analyzed for each subregion separately, none of the subregions had a significant effect. This failure to detect an effect in separate regions suggests either that this measure is not sensitive, that the effects of amphetamine on lever preference ratios were so subtle that dozens of animals are needed to detect them, or both.

During these sessions, we also monitored the rats' locomotor activity as reflected by the movements of their electrical swivels (The data were analyzed using an 8×3×2×2 mixed ANOVA/MANOVA with subregion as between-subjects factor and concentration, trial and direction as within-subjects factors). Although amphetamine injections were unilateral, we did not detect a reliable bias toward ipsiversive or contraversive direction for any subregions. Therefore, ipsiversive and contraversive counts were combined into values referred to as “locomotor activity” (Fig. 1C). Noncontingent amphetamine reliably increased activity when injected into the medial and lateral olfactory tubercle, medial accumbens shell and accumbens core (interaction between effects of subregion and concentration, *F*_14,116_ = 3.25, *P*<0.0002). Noncontingent amphetamine injections into the medial olfactory tubercle increased activity greater than into any other subregion (main effect of subregion, *F*_7,59_ = 8.03, *P*<0.0001). The highest concentration of amphetamine into the medial tubercle increased activity significantly more than administration of the same concentration into any other subregion, while vehicle injections into the medial tubercle did not increase activity more than injections into the other subregions. In rats receiving amphetamine injections into other subregions, activity level was not always parallel with lever pressing. Amphetamine injections into the lateral olfactory tubercle reliably increased activity, but not lever pressing. Similarly, the medium concentration of amphetamine injections into the accumbens core reliably increased lever pressing, but not activity, whereas injection of the highest concentration into the core significantly increased activity, but not lever pressing.

Effects of noncontingent amphetamine administration on responding followed by visual signals were generally similar to the effects of amphetamine on self-administration. Rats learn to self-administer amphetamine into the medial olfactory tubercle and medial accumbens shell more effectively than other subregions [38], although there are some differences between these sites. Amphetamine self-administration into the core is significantly lower than self-administration into the medial shell or medial tubercle in previous studies, whereas responses followed by visual signals facilitated by core injections were equally effective as those of medial shell, but lower than those of medial tubercle. Thus, core injections of amphetamine were more effective in noncontingent administration procedures than self-administration procedures. These findings suggest that the roles of ventral striatal subregions in behavioral interaction with contingent amphetamine (as primary reward) are not identical to their roles in behavioral interaction with unconditioned visual stimuli facilitated by noncontingent amphetamine.

### Experiment 2: Effects of Noncontingent Amphetamine on Lever Pressing Followed by Visual Signals or Tone

**Figure 2:**
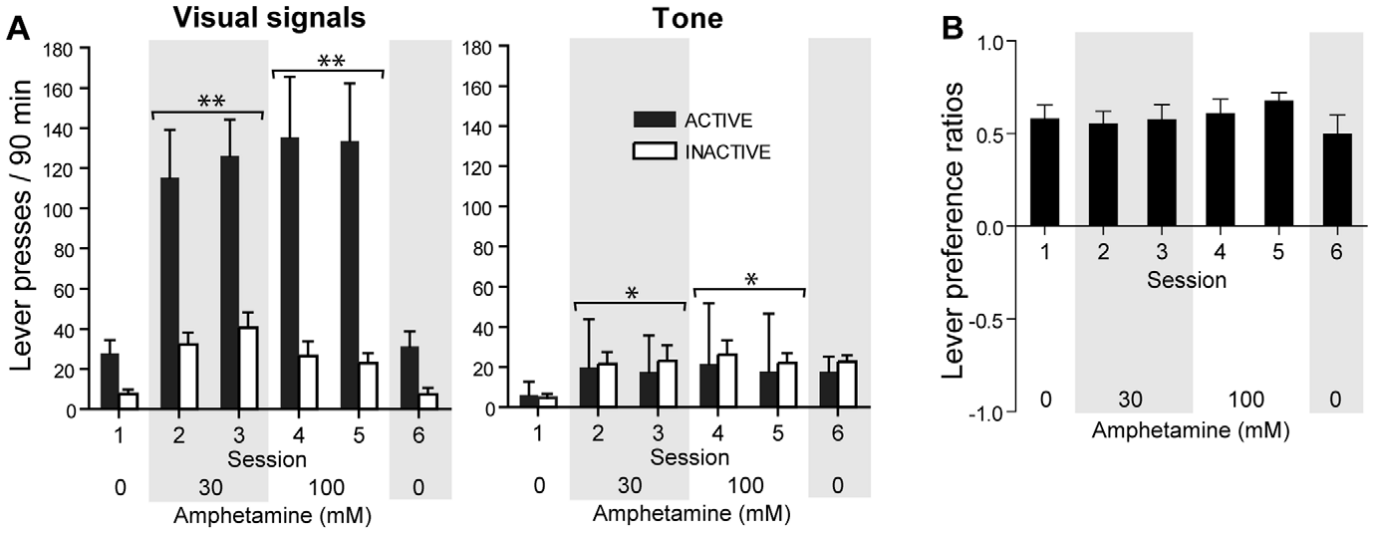
Effects of noncontingent amphetamine on lever responses followed by visual signals or tone. Upon active lever pressing, the visual signal group (n = 8) received an illumination of the cue lamp just above the lever for 1 sec and an extinction of the house lamp for 7 sec, whereas the tone group (n = 8) received a 1 sec tone. Both groups received noncontingent infusions (100 nl per infusion) on a fixed 90-sec interval schedule, just like the groups described in experiment 1 (Fig. 1). A. Lights, but not tone stimuli, support robust lever-pressing in the presence of amphetamine. B. Lever preference ratios of the visual signal group did not reliably differ as a function of amphetamine dose. Data are means ± SEM. * *P*<0.05, ** *P*<0.005, significantly greater than vehicle values.

The behavioral literature suggests that mere presentation of visual stimuli that have not been conditioned with any primary reward can be rewarding in rats and other laboratory animals [14], [15], [16], [17]. This may not be true for aural stimuli like tones. Although tones have been used extensively in research as a conditioned stimulus, we do not know of any report that mere presentation of unconditioned tones is rewarding in rats. Thus, we examined whether noncontingent administration of amphetamine into the medial olfactory tubercle facilitates responding followed by a tone (2900 Hz, a frequency commonly used in rat studies) as well as visual signals. In sessions 2–5 in which the rats received noncontingent amphetamine, the visual signal group markedly increased lever pressing (Fig. 2A; a significant main concentration effect, *F*_2,6_ = 13.38, *P* = 0.006, using a repeated measures ANOVA/MANOVA with concentration (0, 30 and 100 mM) and lever (active and inactive) and session (two for each concentration)), whereas the tone group marginally increased lever pressing (a significant main concentration effect, *F*_2,14_ = 4.84, *P* = 0.025). The visual signal group discriminated between the active and inactive levers (a significant main lever effect, *F*_1,7_ = 10.70, *P* = 0.014, with no significant lever x concentration interaction, *F*_2,6_ = 2.86, *P* = 0.13), whereas the tone group responded on the active lever variably as reflected by large error bars compared to those of the inactive lever and failed to discriminate between the levers.

Even during vehicle sessions, the visual signal group responded on the active lever more than the inactive lever, whereas the tone group did not (a significant interaction between effects of group and lever, *F*_1,7_ = 12.79, *P* = 0.0030, using a mixed ANOVA/MANOVA with group and lever and session). This result is consistent with the previous finding that mere presentation of unconditioned visual stimuli can be rewarding in rats [15], [16]. Rats' preference for the active lever, which delivered visual signals, over the inactive lever did not reliably change with amphetamine administration (Fig. 2B). This result is consistent with the experiment 1 finding that even though lever preference for the active lever increased with the data from all injection sites combined together, no analysis done for each site separately yielded a significant effect on lever preference.

These results have two important implications. First, visual signals are inherently rewarding (positively salient or motivating) to rats, but tones are not (for discussion on related issues, see the 3^rd^ paragraph on experiment 6 and the 2^nd^ paragraph of the General Discussion section); and amphetamine administration into the vicinity of the medial olfactory tubercle enhances actions associated with salient stimuli. Secondly, amphetamine administration into the medial tubercle does not seem to primarily affect the perception of the value of visual signals, because the effect of amphetamine administration on lever preference seems to be minuscule. Although it has to be demonstrated in additional experiments, the finding that amphetamine administration into the medial olfactory tubercle increased both levers may be explained by the notion that visual stimuli reinforce exploration rather than specific responding during enhanced dopamine transmission in the vicinity of the medial tubercle.

### Experiment 3: Effects of Delayed Visual Signal Presentation

The learning literature shows that temporal contiguity between responding and reward presentation is critical for the acquisition and maintenance of rewarded responding. To determine whether amphetamine's capacity to facilitate responding was controlled by visual signals, we examined the effects of temporal contiguity of visual signal presentation on lever pressing.

**Figure 3:**
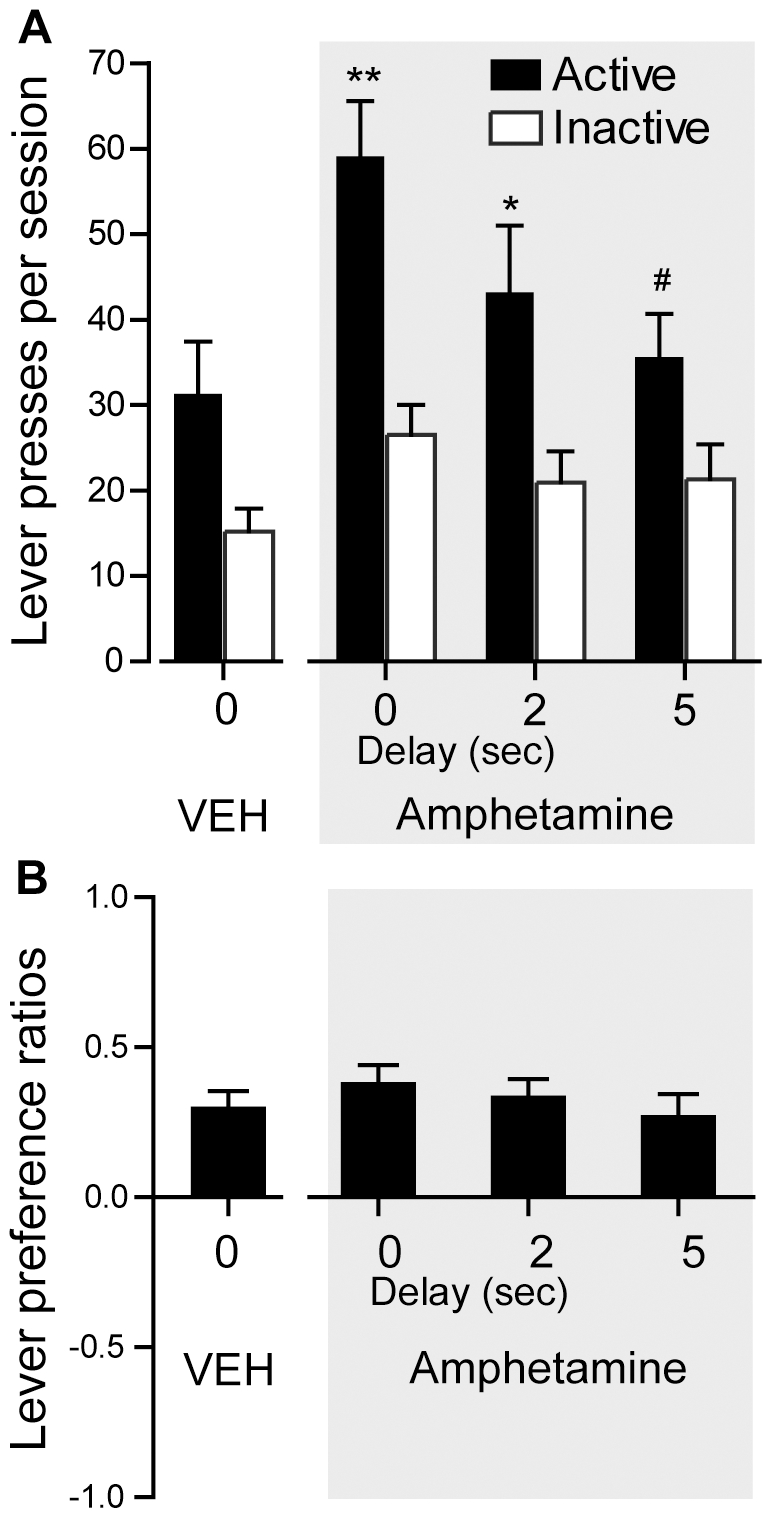
Effects of the delayed visual signal presentation upon lever pressing. Following a noncontingent vehicle (VEH) session, rats received noncontingent administration of amphetamine (30 mM; 78 nl per infusion) into the medial olfactory tubercle. The data (n = 13) are means with SEM. A. Delayed presentation of visual signals decreased active lever presses, while not reliably influencing inactive lever presses. ** *P*<0.001, significantly greater than its inactive lever presses and the active lever presses of the 2- and 5-sec delay sessions. * *P*<0.05, significantly greater than its inactive lever presses and the active lever presses of the 5-sec delay session. ^#^ *P*<0.001, significantly greater than its inactive lever presses. B. Lever preference ratios did not reliably differ as a function of delay.

Delayed presentation of visual signals decreased active lever pressing facilitated by amphetamine, but did not reliably influence inactive lever presses (Fig. 3A; a significant delay x lever interaction, *F*_2,11_ = 4.95, *P* = 0.029). However, analysis of lever-preference ratios did not yield a reliable effect (Fig. 3B). In any case, these results suggest that the temporal contiguity between lever presses and light presentations is critical for intra-tubercle amphetamine injections to facilitate the interaction.

### Experiment 4: Effects of Intraperitoneal Administration of Amphetamine

**Figure 4:**
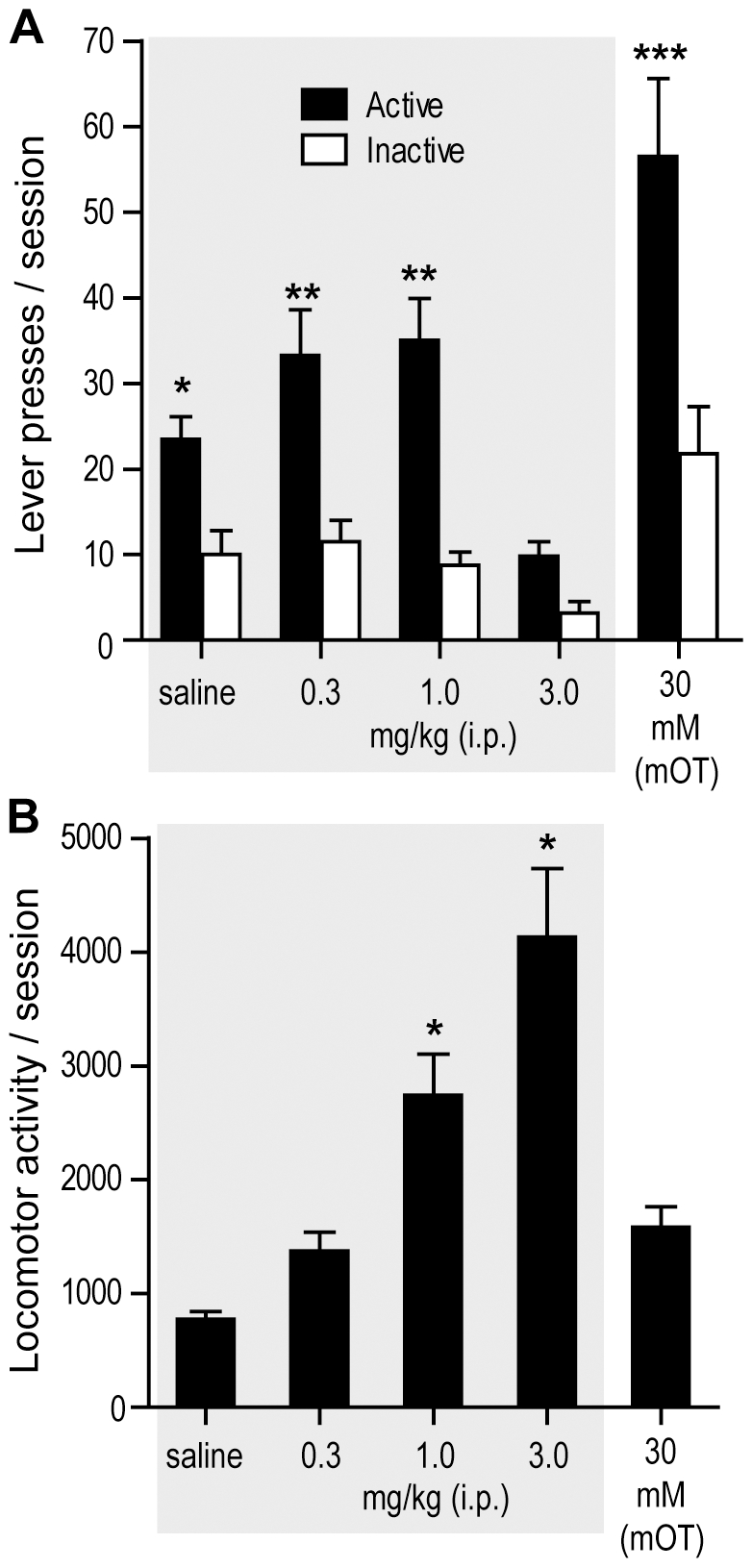
Effects of intraperitoneal administration of amphetamine. Rats (n = 13) received systemic injections of vehicle or amphetamine (0.3, 1, and 3 mg/kg, i.p.) just prior to each session, except that in the last session, they received noncontingent intra-tubercle amphetamine (30 mM; 78 nl per infusion). A. Systemic 0.3 and 1 mg/kg amphetamine slightly increased active lever presses, whereas the 3 mg/kg dose markedly decreased lever presses. ^*^ *P*<0.001, significantly greater than its inactive lever presses and the active lever presses of the 3 mg/kg session. ^**^ *P*<0.005, significantly greater than its inactive lever presses and the active lever presses of the saline session. ^***^ *P*<0.0005, significantly greater than its inactive lever presses and the active lever presses of all other sessions. B. Systemic 1 and 3 mg/kg amphetamine increased locomotor activity. ^*^ *P*<0.005, significantly greater than the values of the saline, 0.3 mg/kg and intra-tubercle sessions.

As mentioned in the introduction, systemic administration of amphetamine in rats markedly increases locomotor activity without facilitating exploration [18], [34]-[36]. We examined whether systemic administration of amphetamine increases locomotor activity and facilitates lever pressing rewarded by visual signals. Systemic doses of 0.3 and 1 mg/kg amphetamine slightly increased lever pressing, whereas the highest does (3 mg/kg) clearly decreased lever pressing (Fig. 4A). Intra-tubercle injections of amphetamine significantly increased lever pressing. These observations were confirmed by a significant interaction between effects of injection manipulation and lever, *F*_4,9_ = 9.08, *P* = 0.0032, using a 4×2 within-subjects design ANOVA/MANOVA with injection manipulation (0, 0.3, 1, and 3 mg/kg, i.p. and intra-tubercle 30 mM amphetamine) and lever.

Amphetamine manipulations had dissimilar effects on locomotor activity. Systemic administration of amphetamine increased locomotor activity in a dose-dependent manner (Fig. 4B; a significant main manipulation effect, *F*_4,9_ = 11.50, *P* = 0.0014, using one-way within-subjects design MANOVA with injection treatment). In particular, the highest dose (3 mg/kg), which decreased lever pressing, markedly increased activity during testing. Intra-tubercle injections of amphetamine, which significantly increased lever pressing, moderately increased activity at levels comparable with the lowest dose of systemic amphetamine (0.3 mg/kg); these effects were not statistically significant.

These results show striking dissociations between lever pressing and locomotor activity and suggest that lever pressing is not a byproduct of heightened locomotor activity or “general” arousal. In other words, lever pressing facilitated by amphetamine into the medial tubercle is not readily explained by the drug's general effects on locomotor activity. Thus, systemic administration of amphetamine may activate multiple behavior facilitation systems, which may interfere with each other or operate independently.

### Experiment 5: Effects of Schedule of Amphetamine Administration

The behavioral literature suggests that intermittent deliveries of small amounts of food, as opposed to a single delivery of a large amount of food, result in “scheduled induced behavior,” in which rats and other animals increase seemingly non-functional responses [43]. Skinner [44] referred to such responses as “superstitious,” reasoning that animals learn false contingencies between responses and food delivery. To determine whether intermittent schedules of amphetamine administration are important for increasing lever pressing rewarded by visual signals, we compared two schedules of amphetamine administration. One is our standard 90-sec fixed interval schedule involving 60 infusions (78 nl per infusion) in the 90 min session, while the other is a “continuous” schedule involving 360 infusions (13 nl per infusion) per session (the shortest increment of delivery that our infusion pump could permit). Thus, both schedules delivered the same amount of amphetamine into the medial olfactory tubercle in the 90-min session. We hypothesized that if intermittent injections are responsible for increasing lever pressing, the two schedules should result in different levels of lever pressing.

**Figure 5:**
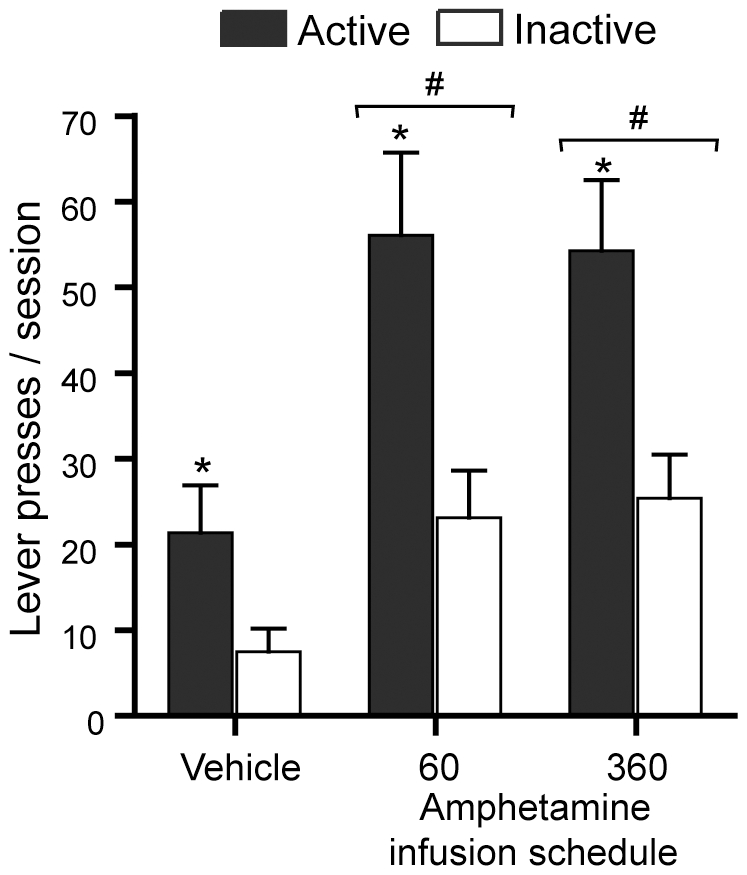
Effects of schedule of amphetamine administration. Rats (n = 8) received amphetamine into the medial olfactory tubercle on two different fixed-interval schedules, 60 infusions (78 nl per infusion) and 360 infusions (13 nl per infusion) in the 90 min session. The data are means with SEM. * *P*<0.01, significantly greater than the values of the inactive lever. # *P*<0.005, significantly greater than the values of the vehicle session.

Lever presses increased when rats received amphetamine in the sessions with the 60 or 360 infusion schedules compared to lever presses with vehicle infusions (Fig. 5). Lever presses between the 60 and 360 infusion schedules did not reliably differ. These observations were confirmed by a significant schedule x lever interaction *F*_1,7_ = 4.91, *P* = 0.024, using a repeated measures ANOVA/MANOVA with schedule manipulation (vehicle and 60 and 360 infusions of amphetamine) and lever.

These results suggest that increased lever pressing rewarded by visual signals persists even with a nearly continuous schedule of amphetamine administration, a finding consistent with previous studies using noncontingent administration of nicotine [39]. Therefore, the lack of effect following systemic administration of amphetamine (experiment 4) is not readily explained by the injection schedule. This conclusion is also consistent with our preliminary data that intermittent intravenous administration of amphetamine did not increase lever pressing rewarded by visual signals (Suto, Shin & Ikemoto, unpublished observation).

### Experiment 6: Effects of Contingent Offset of Visual Signals

We previously suggested that amphetamine injected into the medial olfactory tubercle is rewarding based on the finding that rats learn to increase lever pressing followed by amphetamine injections [38]. Because this self-administration study employed visual signals accompanied with amphetamine injections, this increased lever pressing may have been due to the contingency of visual signals rather than the amphetamine administration. In that earlier study, amphetamine injections were accompanied by the offset of the light stimulus, inverse to the paradigm used in the current study. Here, we sought to determine if rats will lever-press to obtain removal of illuminated visual signals, and if this responding is enhanced by noncontingent amphetamine.

**Figure 6:**
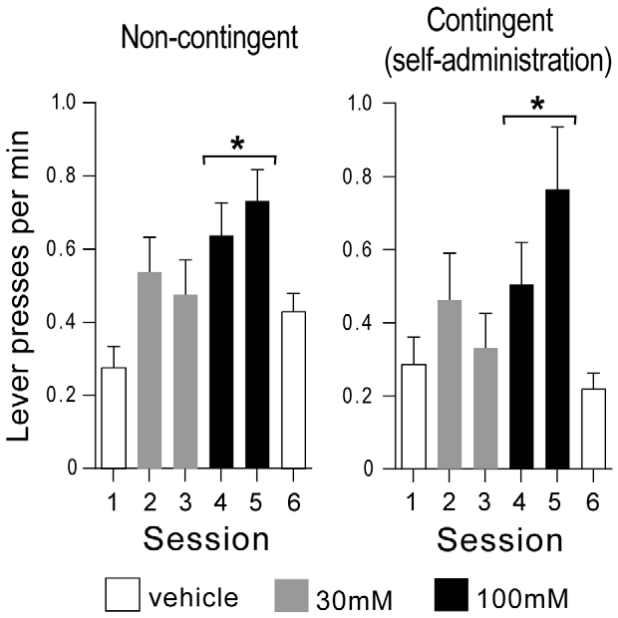
Effects of contingent offset of visual signals. The data are mean lever press rates per session with SEM. The left graph shows the data from the rats (n = 7) that received amphetamine or vehicle infusions (78 nl per infusion) into the medial tubercle on noncontingent schedules (Table 1). A response on the lever extinguished a cue light just above the lever for 5 sec; additional lever presses had no programmed consequence until 5 sec passed, at which time the cue light was reinstated. Amphetamine was delivered on fixed interval schedules; amphetamine administration rates were obtained from median rates of the corresponding sessions of the self-administration group shown in the right graph (n = 10), adopted from Ikemoto et al. [38]. * *P*<0.05, significantly greater than vehicle values.

The noncontingent administration of amphetamine into the medial olfactory tubercle significantly increased lever pressing followed by removal of the illuminated visual cue just above the lever (Fig. 6; a significant main concentration effect, *F*_2,12_ = 6.01, *P* = 0.016 using a repeated ANOVA with 3 concentrations and 2 sessions for each concentration as within-subjects factors). The levels of lever pressing obtained with noncontingent schedules were strikingly similar to those obtained with the contingent schedules [38]. These results suggest that removal of illuminated visual signals is an effective reward for facilitating responding with intra-tubercle amphetamine.

In light of the findings of experiments 1–6, visual sensation involving either the onset or offset of a light stimulus, but not the light per se, appears to facilitate responding in conjunction with intra-tubercle amphetamine. These findings resonate with Kavanau's view that animals learn to increase responding “to exercise control over the stimulus” [45]. After studying wild mice in captivity, Kavanau suggested that the ability to exercise control over a stimulus, rather than the nature of that stimulus, is crucial for the reinforcing effects of such stimuli ranging from visual cues to wheel running to brain stimulation. Because our rats did not respond to aural stimuli, the nature of the stimulus seems to be critical. However, it is still possible that visual signals are rewarding as the subjects of control rather than for their visual content. This question should be addressed by future investigation.

### Experiment 7: Effects of Contingent Administration of Amphetamine without Visual Signals

**Figure 7:**
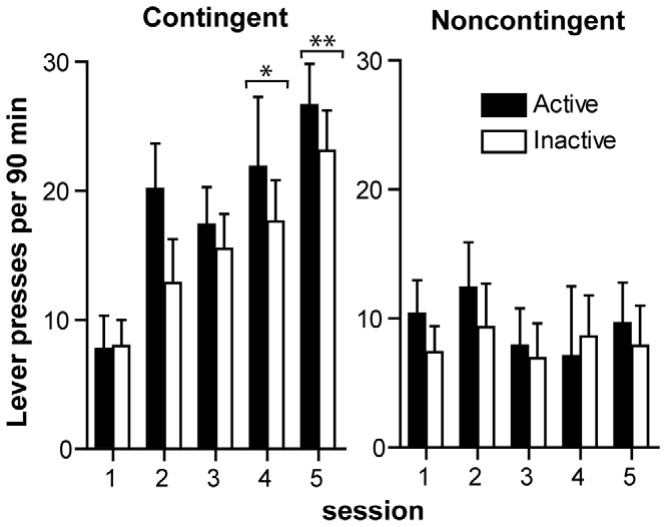
Effects of contingent administration of amphetamine without visual signals. The contingent group (n = 8) received an infusion (78 nl) upon active lever pressing, and the noncontingent group (n = 8) noncontingently received similar amounts of infusions. Both groups received vehicle in session 1 and amphetamine in sessions 2-5, but no visual signals throughout the experiment. The data are mean lever presses per session with SEM. * *P*<0.05, ** *P*<0.001, significantly greater than vehicle values in session 1.

This experiment was designed to determine whether the administration of amphetamine into the medial olfactory tubercle is *reinforcing* in the sense that it would facilitate associative learning (memory consolidation processes associated with the administration event), leading to increases in drug-associated lever pressing [46]–[48]. We examined whether contingent administration of amphetamine into the medial olfactory tubercle without visual signals increased lever pressing. In session 1, when rats received vehicle injections, both contingent and noncontingent groups responded little on both levers and did not discriminate between them. In sessions 2–5, when vehicle was replaced with amphetamine, the contingent group gradually increased responding on both levers over the sessions, but the noncontingent group did not (Fig. 7; a significant interaction between effects of group and session, *F*_4,11_ = 5.40, *P* = 0.012, using a mixed ANOVA/MANOVA with 2 groups as a between-subjects factor and 2 levers and 5 sessions as within-subjects factors). Both groups failed to discriminate between the active and inactive levers, although there was a trend toward the active lever with group collapsed together (a main lever effect, *F*_1,14_ = 3.83, *P* = 0.071).

The significant increases in lever pressing by the contingent group are most likely due to the contingency between lever pressing and amphetamine administration, rather than the general effects of amphetamine on locomotor activity or other factors. This analysis is supported by the finding that the noncontingent group undergoing the identical procedure (except injection contingency) and similar amphetamine amounts did not increase lever pressing. The noncontingent group of rats was subsequently able to discriminate between the two levers in experiment 5, ruling out the possibility of deficits in ability to respond to amphetamine or distinguish the levers. The present experiment's results are consistent with the findings of experiments 2–4 that noncontingent administration of amphetamine into the medial tubercle does not increase lever pressing unless it is followed by visual signals. Thus, this experiment suggests that amphetamine administration into the medial olfactory tubercle or in its vicinity is reinforcing. The involvement of the medial olfactory tubercle in reinforcement is consistent with the previous finding that cocaine injections into the medial olfactory tubercle induce conditioned place preference [37], [49]. The testing of place conditioning was done in the absence of any drug, suggesting that the place preference effect depends on learned associations between drug-paired environmental stimuli and drug-induced states. However, the present experiment suggests that without contingent cues, it is difficult, though not impossible, for rats to guide their actions precisely for intracranial delivery of the drug reward, which does not have its own sensory attributes.

### Experiment 8: Effects of Dopamine Receptor Antagonists

**Figure 8:**
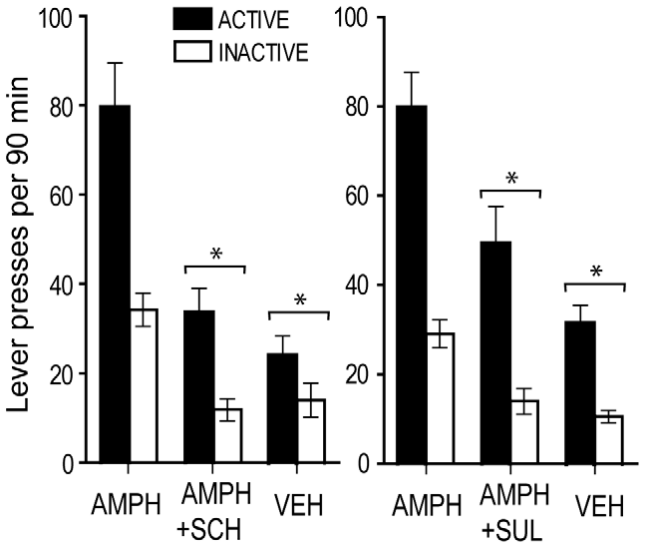
Effects of dopamine receptor antagonists. Rats (n = 9) were presented with visual signals upon active lever pressing while receiving noncontingent injections (100 nl) of amphetamine, amphetamine plus receptor antagonist, and vehicle into the medial olfactory tubercle over 3 sessions. When rats received vehicle or amphetamine (AMPH; 30 mM) mixed with the D1 receptor antagonist SCH 23390 (SCH; 1 mM) or the D2 antagonist sulpiride (SUL; 3 mM), lever pressing significantly decreased. The data are means with SEM. * *P*<0.005, significantly lower than the values of amphetamine alone.

We examined the effects of the blockade of dopamine receptors on lever pressing facilitated by noncontingent amphetamine and rewarded by visual signals, using the same instrumental procedure described in experiment 1. Co-administration of SCH 23390 or sulpiride with amphetamine into the medial olfactory tubercle significantly decreased lever pressing (Fig. 8) with a significant main manipulation effect (*F*_2,7_ = 31.29, *P* = 0.0003) and a significant lever x manipulation interaction (*F*_2,7_ = 5.16, *P* = 0.042) for SCH 23390 and a significant main manipulation effect (*F*_2,7_ = 204.83, *P*<0.0001) and a significant lever x manipulation interaction (*F*_2,7_ = 23.43, *P* = 0.0008) for sulpiride. These results confirm that amphetamine's actions in the vicinity of the olfactory tubercle are mediated by dopamine.

It should be noted that lever-preference analyses revealed no reliable change between the manipulations. In other words, co-administration of receptor antagonists decreased responding on both levers, a finding consistent with the notion that under heightened dopaminergic activity in the medial olfactory tubercle, visual stimuli may reward exploration rather than specific responding.

### Overview

We found that when amphetamine was administered into the medial olfactory tubercle, visual signals that had not been paired with primary rewards gained marked control over rats' actions. Amphetamine's ability to facilitate rats' actions was diminished when these actions were followed by non-salient stimuli or visual signals presented without temporal contiguity. These findings suggest that visual signals play a critical role in facilitating action. Since the amphetamine enhanced lever-pressing was reduced by co-administration of dopamine receptor antagonists, it appears that dopamine receptors mediate amphetamine's ability to facilitate behavioral interaction with visual signals. However, it is intriguing that noncontingent amphetamine injections into the medial olfactory tubercle increased both active (that delivered visual signals) and inactive levers in a similar proportion from their baselines. This effect on inactive lever pressing does not appear to be explained by amphetamine's general effects on locomotor activity, because without visual signals, responding on neither lever increased. In addition, co-administration of dopamine receptor antagonists did not selectively reduce active lever presses, but instead reduced responding on both levers. These findings are consistent with the variation-selection hypothesis of striatal functional organization [50] that enhanced dopaminergic transmission in the medial ventral striatum, including the medial olfactory tubercle, elicits arousal that increases the vigor and variation of approach-type behavior in response to salient stimuli. In this context, dopamine transmission in the more dorsolateral parts of the striatal complex may play a key role in selecting approach-type actions. The present findings on amphetamine likely also apply to cocaine, since we previously observed that intra-tubercle cocaine administration facilitated lever pressing followed by visual signals (described in pp. 56–57 of [50]).

Our data, especially the tone-visual signal comparison data (experiment 2), suggest that amphetamine's ability to facilitate responding for unconditioned stimuli depends on the stimuli's salience. Although we found that visual but not aural signals facilitated seeking with and without amphetamine, it is premature to conclude that aural signals are not salient in rats. For example, these stimuli were not generated from the same location, and a tone generated from the location of the cue light might have been salient and reinforced responding. In addition, since we did not examine a variety of different visual and auditory signals, we do not know if this finding is generalizable to different cues from each sensory modality. For example, auditory stimuli come in different forms, such as tones and clicks, and some may elicit seeking while others might not. Similarly, olfactory stimuli, which are more closely processed by the olfactory tubercle than stimuli from other sensory modalities, may elicit seeking that is facilitated by intra-tubercle psychomotor stimulants. These issues need to be addressed by future research.

### Implications for Distal Stimulus-Controlled Behavior

The present study provides two types of new information crucial for our understanding of the role psychomotor stimulants and dopamine play in distal stimulus-controlled behavior. Firstly, our findings demonstrate that psychomotor stimulants can facilitate action rewarded by distal stimuli that have not been conditioned with primary rewards. We found that selective amphetamine administration into the ventral striatum facilitated action rewarded by visual signals that had not been conditioned with primary rewards, and that this amphetamine-facilitated interaction with visual signals was not a byproduct of the drug's general effects on locomotor activity. Thus, while it was thought that psychomotor stimulant administration enhances action associated with conditioned distal stimuli, which has acquired the motivational properties of primary rewards such as nutrients or drug rewards [1], [3], [6], [12], our findings suggest that psychomotor stimulants also play a key role in stimulus-controlled behavior involving unconditioned distal stimuli. In addition, our results suggest that within the ventral striatum, the medial olfactory tubercle and medial shell and core, but not the lateral tubercle or lateral shell, increase behavioral interaction with unconditioned distal stimuli. The vicinity of the medial olfactory tubercle appears to be most responsive to this function of amphetamine.

Current view on the striatal organization is that the dorsal and ventral striatum have the same basic structure [51], [52], and medio-lateral, rather than dorsoventral, topographical inputs and outputs define functional differences of the striatal complex [53], [54]. In this light, we discuss whether the same ventral striatal mechanisms are responsible for facilitation of behavior with both conditioned and unconditioned stimuli. Mediolateral topography of the inputs and outputs in relation to the nucleus accumbens-olfactory tubercle complex may be responsible for similarities and differences in behavioral functions implicated within each and between the two structures. Indeed, we previously found that the medial part of the ventral striatum, including the medial olfactory tubercle and medial shell, is more responsive than the lateral part of the ventral striatum including the lateral tubercle and lateral shell to rewarding and arousing effects elicited by injections of psychomotor stimulants (cocaine, amphetamine and MDMA) into these subregions [37], [38], [42], [55]. Therefore, differential behavioral effects of psychomotor stimulants on self-administration and locomotion may be roughly correspond to mediolateral topography of ventral striatal connectivity [50]. It is currently unclear whether the accumbens core should be considered as a medial structure. As mentioned above, the core is not as responsive to amphetamine in intracranial self-administration procedures as the medial shell and medial tubercle [38], while the core is as responsive as the shell to noncontingent administration of amphetamine in behavioral interaction with contingent visual signals. In an open field, we did not detect reliable difference in facilitating locomotion and rearing between the medial olfactory tubercle, medial shell and core [42]. If mechanisms are the same, amphetamine injections into the vicinity of the medial olfactory tubercle would be most effective in facilitating behavior with conditioned stimuli. However, given some information that the accumbens core is more important in conditioned behavior than the medial shell [56]–[58], the core may be more responsive to noncontingent amphetamine with conditioned incentive stimuli.

### Implications for Drug Reward

Our findings suggest that some distal stimuli (such as a light turning on or off) that are weakly rewarding alone become powerful instigators of action in the presence of addictive drugs. This notion has important implications for understanding the mechanisms of drug reward. Traditional models of drug self-administration rest on the assumption that behavioral response preceded by drug administration is *reinforced* by the direct pharmacological actions of the drug, and contingent and contiguous drug delivery is thought to be essential for the acquisition of drug self-administration. While we found evidence for this (Fig. 7), we also found that drug-seeking in the absence of visual signals was much weaker than when such signals were present (light turning on or off). Thus, the interaction between drugs and sensory cues could be critically important for understanding the acquisition of drug-taking habit, leading to addiction.

The importance of this interaction between drugs of abuse and salient sensory cues for the acquisition of habitual drug-taking may be less applicable in the human context of systemic administration, since we found that systemic administration of amphetamine was not as effective as intra-tubercle administration in enhancing behavioral interaction with visual signals. However, this may also depend on the drug. Robbins evaluated the effects of systemic administration of four different psychomotor stimulants on conditioned reinforcement in rats [59]. Pipradol and methylphenidate increased responding for conditioned stimuli, while amphetamine and nomifensine did not. Therefore, administration of pipradol or methylphenidate may facilitate behavioral interaction with unconditioned visual signals or other salient distal stimuli. Although it is currently unclear how these psychomotor stimulants differently facilitate stimulus-controlled behavior following systemic administration, pipradol and methylphenidate may be able to more selectively activate the mesolimbic dopamine system than other stimulants.

This notion of the interaction between drugs of abuse and salient sensory cues is also potentially important for understanding tobacco abuse. Caggiula and colleagues found that noncontingent intravenous nicotine administration (intermittent or continuous throughout the session) facilitates lever pressing reinforced by unconditioned visual signals in rats [39], [40]. Contingent administration of nicotine without visual signals faintly reinforces lever pressing, while the co-presentation of visual signals with nicotine makes lever pressing much more vigorous. Our findings build on these nicotine data in two ways. Although nicotine was thought to uniquely facilitate behavioral interaction with unconditioned visual stimuli, these behavioral effects appear to be elicited by other drugs of abuse, including amphetamine (present study) and cocaine [50]. In addition, our finding that this type of seeking is mediated by the dopaminergic mechanisms of the ventral striatum suggests that it may also partly mediate nicotine-driven seeking for unconditioned visual signals. Nicotine receptors are found in the ventral tegmental area [60], which projects to the medial ventral striatum [50]; nicotine administration is known to activate dopaminergic projections to the ventral striatum [61] via the ventral tegmental area [62], [63], leading to self-administration or conditioned place preference [64]–[68]. Thus, nicotine's ability to facilitate behavioral interaction with unconditioned stimuli may be mediated by the mesolimbic dopamine system.

In summary, the present study provides evidence that amphetamine injection into the ventral striatum, particularly the vicinity of the medial olfactory tubercle, facilitates behavioral interaction with unconditioned visual signals, and this effect appears to be mediated by dopamine transmission. This study reinforces the notion that dopamine is involved in distinct functions depending on the subregion within the striatal complex. The most ventromedial part of the striatal complex appears to be important for facilitating behavioral interaction with unconditioned distal stimuli, a process that may both compete with and complement functions such as action-outcome and stimulus-response processes mediated by more dorsolateral parts of the striatal complex.

## Materials and Methods

### Animals

One hundred twenty-eight male Wistar rats (Harlan, Dublin, Virginia; 270–350 g at the time of surgery) were used. The colony room was maintained at a constant temperature and humidity on a reverse 12 h dark 12 h light cycle (8:00 AM off). Food and water were freely available except during testing (90 min or less). The rats were experimentally-naive prior to the start of the surgeries described below. The procedures were approved by the Animal Care and Use Committee of the NIDA Intramural Research Program and were in accordance with National Institutes of Health guidelines.

### Surgery

Rats were stereotaxically implanted with permanent unilateral guide cannulae (24 gauge) under sodium pentobarbital (31 mg/kg, i.p.) and chloral hydrate (142 mg/kg, i.p.) anesthesia. Each rat's guide cannula ended 1.0 mm above one of eight target regions. Cannulae for medial olfactory tubercle and medial nucleus accumbens shell sites were inserted at a 20° angle from the other hemisphere through the midline to minimize diffusion of drug solution to the shell or core, respectively (Fig. 1A). Cannulae were inserted vertically for injections in other regions. The incisor bar was set at 3.3 mm below the interaural line. The stereotaxic coordinates were 2.0 mm anterior to bregma (A), 2.0 mm lateral to the midline (L), and 8.2 mm ventral to the skull surface (V)(measured along the trajectory of the angled cannula) for medial tubercle placements; A2.0, L1.3, V6.5 for dorsomedial shell placements; A2.0, L1.6, V7.2 for ventromedial shell placements; A2.0, L2.5, V8.4 for lateral tubercle placements; A2.0, L2.3, V7.7 for ventral shell placements; A2.0, L1.9, V6.6 for core placements; A0, L2.6, V4.1 for medial caudate putamen; A0, L3.8, V4.5 for lateral caudate putamen. Each cannula was subsequently anchored to the skull by four stainless steel screws and dental acrylic. Rats were housed singly to prevent other rats from chewing the implant after the surgery, which was followed by a minimum of seven days of recuperation before the start of experimentation.

### Drugs

d-Amphetamine, the D1 receptor antagonist R(+)-SCH 23390, and the D2 receptor antagonist S(−)-sulpiride (Sigma, St. Louis, Missouri) was dissolved in an artificial cerebrospinal fluid consisting of 148 mM NaCl, 2.7 mM KCl, 1.2 mM CaCl^2^, and 0.85 mM MgCl^2^ (pH adjusted to 7.4). For systemic injections, d-Amphetamine was dissolved in 0.9% sterile saline.

### Apparatus

Each rat was placed individually in the operant conditioning chamber (30×22×24 cm; Med Associates, St. Albans, VT) equipped with two retractable levers (45 mm wide ×2 mm thick, protruding 20 mm from the wall) below cue lights on a side wall (Fig. 1B) and a standard tone generator (2900 Hz, Sonalert, Med Associates). An injection cannula was inserted and secured into the guide cannula, which was connected by polyethylene tubing to a micropump consisting of a drug reservoir and step motor [69], which hung a few millimeters above the rat's head. When activated, the micropump's step motor turned its shaft in six or eight incremental steps (9° per step) over five sec, driving its threaded shaft into the drug reservoir and, in turn, pushing a 78- or 100-nl volume, respectively, out of the reservoir into the brain.

### Experiment 1: Effects of Injection Sites on Lever Pressing

We examined eight subregions within the ventral and dorsal striatum for effects of noncontingent administration of amphetamine on lever pressing followed by visual signals. Each rat received noncontingent amphetamine administration into one of the subregions in the striatal complex: the medial olfactory tubercle, lateral tubercle, dorsomedial and ventromedial accumbens shell, lateral shell, accumbens core, and medial and lateral caudate putamen (Fig. 1A). In the testing chamber, two levers were available for pressing (Fig. 1B). Upon an active lever press, rats were presented with an illuminated cue light just above the lever for 1 sec and the extinction of the house light for 7 sec, during which lever pressing was counted, but produced no programmed consequence. Responding on the inactive lever had no programmed consequence throughout the session. The left-right locations of the active and inactive levers were counterbalanced among the rats; the assignment of active and inactive functions between the levers remained the same for each rat throughout the experiment. In addition, the number of lever presses required to produce visual signals increased by 1 every 10 stimulus presentations that the rat earned, to facilitate differential responding between the two levers. Rats received vehicle in sessions 1 and 6, 30 mM amphetamine in sessions 2 and 3 and 100 mM amphetamine in sessions 4 and 5. Infusions (100 nl each) were delivered on a 90-sec fixed interval schedule. One of the groups received vehicle injections into the medial olfactory tubercle throughout the experiment. Each session lasted 90 min and sessions were separated by one day.

### Experiment 2: Effects of Noncontingent Amphetamine on Lever Pressing Followed by Visual Signals or Tone

We examined whether the presentation of a tone has similar effects as presentation of a visual signal on lever pressing facilitated by intra-tubercle amphetamine. Each rat received noncontingent amphetamine administration into the medial olfactory tubercle on a 90-sec fixed interval schedule. Rats in the visual signal group were tested with the instrumental procedure described above for experiment 1. Rats in the tone group were placed in the same chambers as the visual signal group. To avoid an effect of illumination change, the light condition for this group was not altered; the house lights remained illuminated throughout the sessions. The rats were played a standard tone used in behavioral conditioning (2900 Hz) for 1 sec upon an active lever press, followed by a 7-sec time-out period, during which lever pressing was counted but produced no programmed consequence. The speakers for tone generation were located immediately below the active lever. For both groups, responding on the inactive lever had no programmed consequence throughout the session. The left-right locations of the active and inactive levers were counterbalanced among the rats; the assignment of active and inactive functions between the levers remained the same for each rat throughout the experiment. As in experiment 1, the number of lever presses required to produce a visual signal or tone increased by 1 every 10 stimulus presentations, and rats received vehicle in sessions 1 and 6, 30 mM amphetamine in sessions 2 and 3 and 100 mM in sessions 4 and 5. Each session lasted 90 min and sessions were separated by one day.

### Experiment 3: Effects of Delayed Visual Signal Presentation

Rats received 30 mM amphetamine infusions (78 nl per infusion) on a 90-sec fixed interval schedule throughout the experiment. A response on the active lever illuminated the cue light for 1 sec just above the lever and extinguished the house light for 1 sec, followed by a 7 sec timeout during which lever pressing was counted, but produced no programmed consequence. To keep the amount and pattern of visual signals the same among the three visual signal conditions described below, the offset of the house light did not correspond to the timeout period in this experiment, unlike experiment 1. Instead, the house lights were turned off upon active lever pressing, to make the visual signal more salient. Responding on the inactive lever had no programmed consequence throughout the session. In this instrumental procedure, rats received vehicle in session 1. Over sessions 2–4, visual signals were presented following delays of 0, 2, and 5 sec upon active lever pressing. The order in which these delay manipulations were tested over the sessions was counterbalanced among the rats. Each session lasted 60 min, and sessions were separated by a day.

### Experiment 4: Effects of Intraperitoneal Administration of Amphetamine

We examined the effects of i.p. administration of amphetamine on lever pressing rewarded by visual signals, using the rats from experiment 3 and the instrumental procedure employed in experiment 1. The rats received vehicle (1 ml/kg 0.9% saline) or one of 3 doses of amphetamine (0.3, 1, and 3 mg/kg) just prior to the start of a session. The order of testing these manipulations over 4 sessions was counterbalanced among rats. After completing these manipulations, the rats received noncontingent 30 mM amphetamine infusions (78 nl) into the medial olfactory tubercle on a 90-sec fixed interval schedule during testing. Each session lasted 90 min. Sessions were separated by a day.

### Experiment 5: Effects of Schedule of Amphetamine Administration

The rats from experiment 7 (described below) were used. Over three sessions, the rats received vehicle on a 90-sec fixed interval schedule in the first session, noncontingent 30 mM amphetamine infusions on a 90-sec fixed interval schedule (60 78-nl infusions), and noncontingent 30 mM amphetamine infusions on a 15-sec fixed interval schedule (360 13-nl infusions). Thus, they received the same amount of amphetamine between the two schedules and were tested with the same instrumental procedure as in experiment 1.

### Experiment 6: Effects of Contingent Offset of Visual Signals

We previously found that rats learn to self-administer amphetamine into the medial olfactory tubercle [38]. In that study, a response on the lever led to both the administration of amphetamine into the medial olfactory tubercle, and the extinction of illuminated cue light above the lever. Here, we examined whether the extinction of the light enables noncontingent administration of amphetamine to facilitate lever pressing.

Because the testing chambers used by Ikemoto et al. [38] were identical to the ones described for this study, here we employed the same behavioral procedure that was used in the published study, except in the case of amphetamine administration contingency, to make these two experiments as comparable as possible. Only one lever was inserted into the testing chamber, and the other lever was retracted (i.e., not available for pressing) throughout the experiment. A lever press led to a 1 sec extinction of the cue light above the lever, followed by a 5 sec timeout during which additional lever pressing had no programmed consequence. To make the offset of the cue light above the lever salient, the house light remained turned off throughout the session in this experiment. Amphetamine or vehicle (78 nl per infusion) was delivered on noncontingent, fixed-interval schedules as described in Table 1, which were derived from mean infusion rates of self-administration sessions in the self-administration study [38]. Each session lasted 90 min, and sessions were separated by a day.

**Table 1:** Amphetamine concentrations and infusion intervals used for each session.

Session	1	2	3	4	5	6
Amphetamine (mM)	0	30	30	100	100	0
Infusion interval (sec)	325	152	193	135	85	323

### Experiment 7: Effects of Contingent Administration of Amphetamine without Visual Signals

Each rat received vehicle (78 nl per infusion over 5 sec) in session 1 and 30 mM amphetamine in sessions 2–5 while in the testing chambers, which were set up as described for experiment 1. A response on either the active or inactive lever retracted both levers, which were reinstated 7 sec after the response. The contingent group received amphetamine infusion upon active lever pressing, and responding on the inactive lever produced no infusions. The noncontingent group received infusions throughout the experiment on a 193-sec fixed interval schedule (total 27 infusions per session), regardless of lever response. This schedule was designed to mimic the level of amphetamine administration received by the contingent group, which received a mean of 27 infusions in session 5.

### Experiment 8: Effects of Dopamine Receptor Antagonists

We examined the effects of the dopamine D1- and D2-type receptor antagonists SCH 23390 and sulpiride on amphetamine-facilitated lever presses followed by visual signals, using the same instrumental procedure described for experiment 1. Rats received injections (100 nl) of vehicle in session 1. In sessions 2–3, each rat received injections of amphetamine (30 mM) alone and the mixture of 30 mM amphetamine and 1 mM SCH23390. The order of testing these two manipulations was counterbalanced among the rats. In session 4, rats received vehicle again. In sessions 5 and 6, each rat received infusions of 30 mM amphetamine and the mixture of 30 mM amphetamine and 3 mM sulpiride. Again, the order of testing the manipulations was counterbalanced.

### Histology

Upon completion of the experiments, the rats' brains were removed under deep anesthesia induced by pentobarbital (31 mg/kg, i.p.) and chloral hydrate (142 mg/kg, i.p.). They were placed in 10% formalin for a minimum of 2 days prior to sectioning on a cryostat. Frozen coronal sections (40-µm thickness) near the cannula tip were mounted on gelatinized glass slides and stained with cresyl violet. Injection cannulae placements were verified by microscopic examination.

### Statistical Analyses

Data were analyzed with the ANOVA/MANOVA module of Statistica (version 6.1, StatSoft, Inc., Tulsa, OK). When the sphericity assumption examined by Mauchley Sphericity Test was violated for repeated factors, effects of the repeated factors were analyzed by MANOVAs; otherwise ANOVAs were used. When a significant effect was found for a factor with more than two levels, a Newman-Keuls post-hoc test was performed.

## Supporting Information

Figure S1 Ventral striatal injection sites and their effectiveness. Photomicrographs depict representative placements of cannulae for the medial and lateral olfactory tubercle, medial and lateral shell of the nucleus accumbens and accumbens core. Arrows indicate the tips of injection cannulae, while arrow heads indicate the tips of guide cannulae (when they are evident). Coronal drawings on the right show 0.3 mm tips of injection cannulas of the rats used in experiment 1 (excluding dorsal striatal rats) and 8 rats that were used in experiment 2 and treated exactly the same. The color of each rectangle indicates injection site's effectiveness (the sum of the two highest responses on the active lever among the 4 amphetamine sessions) with visual signals. Effectiveness was categorized into 4 levels. Category low (gray; 80 or less) is considered to indicate no enhancement, because when the rats did not receive amphetamine in sessions 1 and 6, 90% of them scored 80 or less. The extent of the ventral striatum, which is filled with median spiny GABAergic neurons, is indicated by light shade. There is no divide between the ventral and dorsal striatum, and small non-striatal components (medial forebrain bundle and ventral pallidum) are found at the dorsal part of the olfactory tubercle just ventral to the accumbens shell.
(4.36 MB TIF)
