# Supplementary figures and images for: Amphetamine Administration into the Ventral Striatum Facilitates Behavioral Interaction with Unconditioned Visual Signals in Rats
Source: PLoS One. 2010 Jan 15;5(1):e8741. doi: 10.1371/journal.pone.0008741 (PMC2806927; doi:10.1371/journal.pone.0008741)

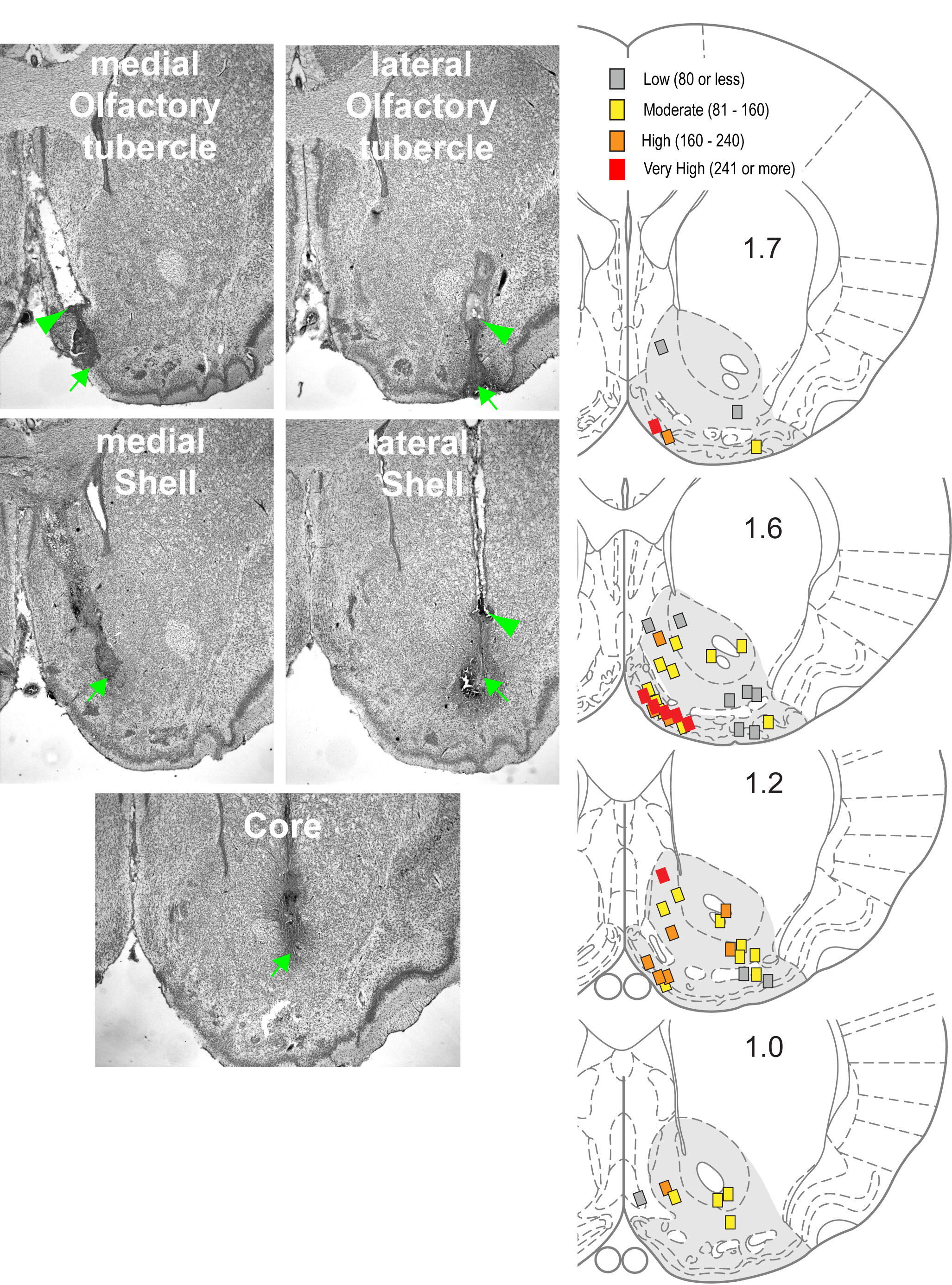

Supplement: Figure S1 — Ventral striatal injection sites and their effectiveness. Photomicrographs depict representative placements of cannulae for the medial and lateral olfactory tubercle, medial and lateral shell of the nucleus accumbens and accumbens core. Arrows indicate the tips of injection cannulae, while arrow heads indicate the tips of guide cannulae (when they are evident). Coronal drawings on the right show 0.3 mm tips of injection cannulas of the rats used in experiment 1 (excluding dorsal striatal rats) and 8 rats that were used in experiment 2 and treated exactly the same. The color of each rectangle indicates injection site's effectiveness (the sum of the two highest responses on the active lever among the 4 amphetamine sessions) with visual signals. Effectiveness was categorized into 4 levels. Category low (gray; 80 or less) is considered to indicate no enhancement, because when the rats did not receive amphetamine in sessions 1 and 6, 90% of them scored 80 or less. The extent of the ventral striatum, which is filled with median spiny GABAergic neurons, is indicated by light shade. There is no divide between the ventral and dorsal striatum, and small non-striatal components (medial forebrain bundle and ventral pallidum) are found at the dorsal part of the olfactory tubercle just ventral to the accumbens shell. (4.36 MB TIF) [file pone.0008741.s001.tif]
